# Crosstalk among Indoleamines, Neuropeptides and JH/20E in Regulation of Reproduction in the American Cockroach, *Periplaneta americana*

**DOI:** 10.3390/insects11030155

**Published:** 2020-03-01

**Authors:** A. S. M. Kamruzzaman, Azam Mikani, Amr A. Mohamed, Azza M. Elgendy, Makio Takeda

**Affiliations:** 1 Department of Agrobioscience, Graduate School of Agricultural Science, Kobe University, 1-1 Rokkodai-cho, Nada-ku, Kobe 657-8501, Japan; asm_kzaman@hotmail.com; 2 Department of Entomology, Faculty of Agriculture, Tarbiat Modares University, Tehran 14115-336, Iran; a.mikani@modares.ac.ir; 3 Department of Entomology, Faculty of Science, Cairo University, Giza 12613, Egypt; aelgendy@sci.cu.edu.eg

**Keywords:** vitellogenesis, *Periplaneta americana*, aaNAT, juvenile hormone, 20-hydroxyecdysone, neuropeptides, biogenic amines, oocytes

## Abstract

Although the regulation of vitellogenesis in insects has been mainly discussed in terms of ‘classical’ lipid hormones, juvenile hormone (JH), and 20-hydroxyecdysone (20E), recent data support the notion that this process must be adjusted in harmony with a nutritional input/reservoir and involvement of certain indoleamines and neuropeptides in regulation of such process. This study focuses on crosstalks among these axes, lipid hormones, monoamines, and neuropeptides in regulation of vitellogenesis in the American cockroach *Periplaneta americana* with novel aspects in the roles of arylalkylamine *N*-acetyltransferase (aaNAT), a key enzyme in indoleamine metabolism, and the enteroendocrine peptides; crustacean cardioactive peptide (CCAP) and short neuropeptide F (sNPF). Double-stranded RNA against *aaNAT* (dsRNA^aaNAT^) was injected into designated-aged females and the effects were monitored including the expressions of *aaNAT* itself, vitellogenin 1 and 2 (*Vg1* and *Vg2*) and the vitellogenin receptor (*VgR*) mRNAs, oocyte maturation and changes in the hemolymph peptide concentrations. Effects of peptides application and 20E were also investigated. Injection of dsRNA^aaNAT^ strongly suppressed oocyte maturation, transcription of *Vg1*, *Vg2*, *VgR,* and genes encoding JH acid- and farnesoate *O*-methyltransferases (*JHAMT* and *FAMeT*, respectively) acting in the JH biosynthetic pathway. However, it did not affect hemolymph concentrations of CCAP and sNPF. Injection of CCAP stimulated, while sNPF suppressed oocyte maturation and *Vg*s/*VgR* transcription, i.e., acting as allatomedins. Injection of CCAP promoted, while sNPF repressed ecdysteroid (20E) synthesis, particularly at the second step of *Vg* uptake. 20E also affected the JH biosynthetic pathway and Vg/VgR synthesis. The results revealed that on the course of vitellogenesis, JH- and 20E-mediated regulation occurs downstream to indoleamines- and peptides-mediated regulations. Intricate mutual interactions of these regulatory routes must orchestrate reproduction in this species at the highest potency.

## 1. Introduction

Oocytes maturation follows massive biosynthesis of vitellogenins (Vgs), the yolk protein precursors, in the fat body. Vgs bind to the Vg receptor (VgR), which belongs to the low-density lipoprotein receptor (LDLR) family, located on the oocyte membrane. After Vgs binding to VgR, they are co-endocytosed from the hemolymph into oocytes via clathrin and Ras-like GTPase Rab protein mechanisms [1,2,3].

Vitellogenesis in insects is regulated by hormones and other signal factors, including ecdysteroids (20E), juvenile hormone (JH), and neuropeptides in unique ways depending on the species [2,4]. JH is a widespread gonadotropin [5], while 20E plays a more important role in evolved insect orders [6]. This simplified view of the process, however, overlooks some important aspects in Vg synthesis and its uptake by oocytes, particularly neuropeptides and monoamines functions. Environmental input may reach the brain, corpus cardiacum (CC), corpus allatum (CA), fat body and follicle cells or oocytes membrane from a variety of origins such as nutritional, seasonal, social and thermal stress conditions. The resorption of unfertilized oocytes occurs in response to environmental stresses, such as starvation and lack of mates to recoup and reinvest nutrients into somatic functions to increase lifespan [7]. In starved *Leptinotarsa decemlineata* females, the CA activity is restrained, and this effect is mediated via nervous pathways from the brain and/or suboesophageal ganglion with no direct influence of the humoral milieu during the initial stages of starvation [8]. Thus, short days and starvation signals may come from independent transduction pathways. Nevertheless, these must be integrated hormonally to achieve a reproductive task, either vitellogenically or non-vitellogenically, but not in between.

Neuropeptides make up a very broad and highly diverse group of molecules that influence diverse physiological and behavioral actions in insects and in animals generally [9]. They act in a variety of modes, such as neurotransmitters, neuromodulators and neurohormones. For an example, Shi et al. [10] identified 23 genes encoding pro-neuropeptides and 51 genes encoding neurotransmitter precursor processing enzymes in the central nervous system of the diamond back moth, *Plutella xylostella*. Some of these signal molecules influence Vg biology. Hemolymph concentrations of FMRFamide increase during vitellogenesis and decease after oviposition [11]. Similarly, injections of neuropeptide F (NPF; including the short form, sNPF) family peptides accelerated egg development in *Locusta migratoria* [12]. The ovary maturing parsins (OMPs) are essential for vitellogenesis in *L. migratoria* and *Schistocerca gregaria* [13,14].

The crustacean cardioactive peptide (CCAP) is a ubiquitous highly conserved cyclic C-amidated nonapeptide, expressed in the central- and stomatogastric nervous systems and midgut of arthropods, including *P. americana*, and acts in regulating digestion [15,16,17]. CCAP can also trigger the motor patterns of ecdysis in *Manduca sexta* [18,19]. CCAP induces the CC to release adipokinetic hormone in locusts [20] and stimulates oviduct contractions in *L*. *migratoria* [21]. However, its role in vitellogenesis is not yet fully addressed. Many allatotropic and allatostatic peptides regulate the CA [22], but the precise mechanism of CA control is still unclear. JH directly influences transcription of *Vg* genes and the subsequent control of Vg biosynthesis in *P*. *americana* [23] with two *Vg* gene isoforms, *Vg1* and *Vg2* [24].

Previously we showed that CCAP and sNPF regulate digestive physiology in the alimentary canal, in both positive and negative feedback loops, and food searching behavior in *P. americana* [16,17]. These neuropeptides convey alarming messages about its nutritional condition to peripheral organs including metabolic center, i.e., the fat body and reproductive organ, since reproduction is a heavy energy consuming task and must be supported by a sufficient nutritional reservoir [1]. The coordination between the reproductive and metabolic organs is vital for survival. Also, the JH/20E route and indoleamine route (see below) have been investigated independently [25,26] and the effects of CCAP/sNPF have been neither investigated directly nor in terms of allatotrophe’s regulatory pathway. Disentangling such a complicated network of pathways acting in insect reproduction is important as it is not always clear to what degree functional data obtained in a given model species can be interpolated to other insect taxa because of the different life-history traits, developmental plasticity and the long evolutionary history of many insect orders [4,22].

The neurohormone melatonin (MEL) occurs in most organisms from protists to higher plants. It mediates circadian rhythms, scavenging of free radicals and bone physiology and reproduction in mammals [27]. Insects produce MEL via a pathway similar to mammals, in which aralkylamine *N*-acetyltransferase (aaNAT) is the key enzyme in indoleamine, catecholamine and phenolamine metabolism [28]. aaNAT is the penultimate enzyme in the synthesis of MEL in vertebrates. This enzyme acetylates a wide range of arylalkylamines. aaNAT transfers an acetyl moiety, a rate-limiting step, to indoleamines and other arylalkylamines including serotonin (5-hydroxytryptamine; 5-HT) to form *N*-acetylserotonin (NAS; normelatonin), a substrate for MEL synthesis [27]. We have shown that the injections of MEL, 5-HT, NAS, tryptamine (TN) and *N*-acetyltryptamine (NATN) significantly modified Vg synthesis and affected two JH-synthesizing enzymes, JHAMT and FAMeT [25,26]. aaNAT is highly expressed in the CNS, midgut and reproductive glands in *P. americana* [25,29]. MEL mediates the switch from parthenogenesis to sexual reproduction in short-day aphids, *Acyrthosiphon pisum* [30]. It may also influence postembryonic developmental processes like molting, eclosion and diapause. MEL treatments stimulated ecdysone release from the prothoracic gland co-incubated with Br-retrocerebral complex of *P*. *americana* via the induced release of prothoracicotropic hormone (PTTH), an ecdysiotropic neuropeptide produced by neurosecretory cells in the brain [31] as well as in *Antheraea pernyi* where MEL stimulated PTTH release to terminate pupal diapause and the injection of dsRNA^aaNAT^ abolished long-day effect [32].

We focused on potential interactions among aaNAT, indoleamines, neuropeptides (CCAP and sNPF) and JH/20E on vitellogenesis and maturation of oocytes in *P. americana* to address whether these routes may form a hierarchy or at least a network of crosstalks with each other or otherwise each may represent an independent pathway from independent input signal(s).

## 2. Material and Methods

### 2.1. Experimental Insects

All experiments were conducted with *P. americana*. Colonies were maintained at 25 °C and 12L:12D photoperiod on an artificial diet (MF; Oriental Yeast, Tokyo, Japan) and water *ad libitum*. Newly molted white roaches were collected daily and kept individually in clear plastic cups (D = 10.0 cm, H = 4.5 cm) with the artificial diet.

### 2.2. RNA Extraction and cDNA Synthesis

Total RNA was isolated from the Brain-retrocerebral complex (brain with the attached paired CA and CC) and fat body with RNAiso plus (Takara Bio Inc., Kusatsu, Japan). Total RNA was dissolved in RNase-free water and the RNA quality was measured for yield and purity using a Nanodrop ND-2000 spectrophotometer (NanoDrop products, Wilmington, DE, USA). RNA integrity was checked on an Agilent 2100 BioAnalyzer (Agilent Technologies, Colorado Springs, CO, USA). The RNA samples were treated with 2 units of DNase I to remove trace amount of genomic DNA. One µg total RNA was incubated at 70 °C for 3 min, and then converted to cDNA for each reaction with ReverTra Ace^®^ reverse transcriptase (Toyobo, Osaka, Japan) according to the manufacturer’s instructions (using an initial and single cycle of 37 °C 15 min, 50 °C for 5 min, 98 °C for 5 min). The synthesized cDNA served as a template for PCR.

### 2.3. Preparation and Injection of dsRNA

The brain-subesophageal ganglion complex was isolated and immediately transferred into liquid N_2_ and total RNA was extracted with the RNAiso Plus reagent (Takara Bio Inc., Kusatsu, Japan). Two hundred fifty nanograms of total RNA and SMART™ RACE cDNA Amplification Kit (BD Biosciences Clontech, Shiga, Japan) were used for synthesis of the cDNA.

Double stranded DNA of a 282-base pair (bp) fragment of *aaNAT* (GenBank accession number: AB106562.1) was amplified by PCR. Primers used to generate template for in vitro transcription contained a T7 promoter (with the sequence TAATACGACTCACTATAGGGAGA, underlined below) at each end. The primers used for amplification were the forward primer: 5′-TAATACGACTCACTATAGGGAGATAATGGCAGTATCCAGAAC-3′; and the reverse primer: 5′-TAATACGACTCACTATAGGGAGAGATATTATGCGCACTTCTAC-3′. The PCR mixture (50 μL) included 4 μL of 50-fold diluted cDNA template, 5 μL (10 pmol) of each primer, 5 μL of 2 mM dNTPs, 25 μL of 2X buffer and 1 μL of KOD FX Neo (Toyobo Co. Ltd., Osaka, Japan). PCR (35 cycles) was performed as follows: denaturation at 98 °C for 10 s, annealing at 65 °C for 30 s, extension at 68 °C for 5 min. The *aaNAT* double-stranded RNA (ds*aaNAT*) was synthesized from purified PCR product by using Ambion’s MEGAscript^®^ RNAi Kit (Thermo Fisher Scientific, Waltham, MA, USA) according to the manufacturer’s protocol. For an experimental group, 32 µg of dsRNA was injected into the abdomen of freshly molted individual females (i.e., 0-days post adult molt). The cockroaches in the control group were injected with an equal volume of H_2_O. After injection, each cockroach was immediately returned to the rearing container. We also injected dsGFP into cockroaches and found no effect on *aaNAT* transcription (results not shown). However, in view of recently reported off-target effects of dsGFP injection (impairing its host’s core biological functions via affecting several genes implicated in central metabolic and developmental processes associated with RNA processing and transport, hormone metabolism, immunity and response to external stimulus and to stress) to control for nonspecific side effects of RNAi [33,34], we favored water as a control for RNAi, alone, as a widely used accepted approach in RNAi studies in insects [35,36].

### 2.4. mRNA Quantification

The sequences of *P*. *americana* targeted genes *aaNAT* (GenBank: AB106562.1), *Vg1* (GenBank: AB034804.1), *Vg2* (GenBank: AB047401.1), *VgR* (GenBank: AB077047.2), *JHAMT* (GenBank: LC164750.1), *FAMeT* (GenBank: LC164751.1) and *Actin* (GenBank: AY116670.1) were retrieved from NCBI (data from our laboratory). The gene-specific primers were designed using Primer 3 Tool. RNAs and template cDNAs were prepared as mentioned above. qPCR was performed with the SYBR^®^ Green Realtime PCR master mix (Toyobo, Osaka, Japan), with the forward and reverse primers shown in Table 1. Cycling parameters were 95 °C for 1 min, 40 cycles programmed for 95 °C for 15 s and 60 °C for 2 min. The acquisition of fluorescence data was performed at the end of the elongation step with Applied Biosystem 7500 real PCR system (Applied Biosystems, Foster City, CA, USA). Initial amount of template was calculated from cDNA standard curve generated for each PCR run. Three independent biological replicates containing three pooled insects for each were run per condition. Relative transcript quantity was calculated using the C_T_ (ΔΔC_T_) method [37]. *Actin* mRNA was used as the reference gene for all samples.

### 2.5. Competitive ELISA

Cockroaches were anesthetized by ice cooling before dissection. The hemolymph extracted from female cockroach was diluted in TBS and after centrifugation (4000× *g*, 4 °C, 15 min), the supernatant was used for ELISA [38]. A CCAP-BSA or sNPF-BSA conjugate was prepared by coupling CCAP or sNPF to BSA with dimethyl suberimidate (Sigma-Aldrich, Buchs, Switzerland). The coating of well plates (Corning Inc., Corning, NY, USA) was prepared with antigen-BSA (0.6 µg/mL per well) in 0.05 M sodium carbonate-bicarbonate buffer (pH 9.0) for 3 h, then plates were blocked with 250 μL of 2% skim milk for 1 h at RT. Standard peptide solutions (0.01–100 nmol/well) or the supernatant of the hemolymph was added in a volume of 50 μL/well. Fifty microliters of the antibody diluted in TBS with 2% skim milk (CCAP 1:9000, sNPF 1:13,000) was subsequently added to each well and the plate was incubated overnight at 4 °C with light shaking. The plates were rinsed three times with TBS containing 0.5% Tween-20 (TBS-Tw) after incubation and were then incubated with 100 μL of the secondary antibody solution containing goat immunoglobulin anti-rabbit IgG labeled with alkaline phosphatase at 1:1000 in TBS at RT for 1 h. After the plates were rinsed three times, 100 μL of substrate solution [1 mg/mL 4-nitrophenyl phosphate disodium salt hexahydrate (Sigma-Aldrich, Gillingham, UK) in 10 mM diethanolamine buffer (Sigma-Aldrich, St. Louis, MO, USA), pH 9.5] was added to each well and incubated for 1 h at RT. The reaction was stopped by the addition of 50 μL of 4 M NaOH and the absorbance was read at 405 nm using a microplate reader (Bio-Rad, Hercules, CA, USA).

### 2.6. CCAP and sNPF Injection into the Hemolymph

Ten pmol CCAP/sNPF (Invitrogen, Carlsbad, CA, USA) in 4 μL of phosphate-buffered saline (PBS; 145 mM NaCl, 1.45 mM NaH_2_PO_4_, 8.55 mM Na_2_HPO_4_, pH 7.5) was injected into the hemocoel of female adults using a Hamilton syringe (Hamilton Company, Reno, NV, USA). They were injected daily from day 0 to day 15 of adult stage. Control insects were injected with 4 μL PBS. The puncture made by the injection was sealed with the instant adhesive, Aron Alpha (Toagosei, Tokyo, Japan).

The effect of CCAP/sNPF on oocyte maturation was evaluated by dissecting the ovaries and measuring oocyte size on days 3, 6, 9, 12 and 15. It also was applied for verifying effects of CCAP/sNPF on ecdysteroid in ovaries on the day 15 and hemolymph at several time points during the experiments. On the last day of the experiment (day 15) ovaries were dissected. The injection of CCAP and sNPF (10^−7^ moles) in 10 μL of PBS were performed to investigate the effect on the transcription of *JHAMT/FAMeT* and *Vgs* and oocyte development.

### 2.7. Immunocytochemistry

Immunocytochemistry was performed on ovaries that were fixed in a Bouin’s solution and kept overnight at 4 °C after dissection in PBS solution, and were then dehydrated and embedded in paraffin. Standard histochemical methods were used for tissue dehydration, embedding in paraffin, sectioning to 8 µm, deparaffinization, and rehydration, and blocking as described earlier [32]. Immunocytochemistry was performed with a Rabbit IgG-Vectastain Elite ABC kit (Vectastain ABC KIT PK-6101). The sections were blocked with 5% (*v*/*v*) normal goat serum in TBS-T (blocking serum) for 30 min at RT, and incubated with rabbit anti-PaVgR primary antibody (diluted 1:100,000 in blocking serum) in a humidified chamber overnight at 4 °C. In control experiments, the primary antibody was replaced with preimmune rabbit serum. After thorough rinsing with TBS-T (3 × 10 min) at RT, the sections were first incubated with a biotinylated secondary antibody (diluted 1:200 in blocking serum) for 1 h at RT, rinsed again with TBS-T (3 × 10 min), and then treated with a horseradish peroxidase-(HRP) labelled avidin-biotin complex (diluted in TBS-T) for 50 min at RT. After the incubation, sections were thoroughly washed with TBS-T (3 × 10 min) and with 0.05 M Tris-HCl, pH 7.5 (1 × 10 min). The peroxidase activity was developed using hydrogen peroxide (0.005%) and 3,3′-diaminobenzidine tetrahydrochloride (DAB, 0.25 mM in 0.05 M Tris-HCl, pH 7.5) as a chromogen. Stained sections were dehydrated and mounted on Bioleit mounting medium (Kouken Rika, Osaka, Japan) and were examined under a BX50F4 microscope (Olympus, Tokyo, Japan).

### 2.8. Measurements of Ecdysteroid (20E) Titer

Ecdysteroid levels were quantified with enzyme immunoassay (EIA), as per Porcheron et al. [39] procedures with modifications. For collection and extraction of hemolymph samples at different time points, 5 μL hemolymph was collected from each female and diluted in 100 μL ice cold methanol. Samples were centrifuged three times and the supernatants were collected. All supernatants from the same sample were combined and dried in vacuum centrifuge. The residue was dissolved in 0.1 M phosphate buffer (pH 7.4) and stored in −20 °C.

Ovaries were dissected in Ringer solution and processed by extraction with ice cold methanol (2 mL), followed by homogenization with a bar sonicator. The resulting homogenates were heated at 60 °C for 10 min, followed by centrifugation at 10,000× *g* for 10 min. The supernatants were collected, and the pellets were re-extracted twice into 1 mL 70% methanol. The supernatants of both centrifugations were joined and dried completely by evaporation in a vacuum centrifuge. For obtaining optimal EIA measurements, apolar lipids were removed from the samples. The pellets were dissolved in 1 mL 70% methanol and 1 mL 100% hexane, followed by mixing, centrifuging and discarding the upper hexane phase. The remaining methanol phase of each sample was divided in two equal halves. Both halves were completely dried in a SpeedVac concentrator. One of them was dissolved in the sample buffer for EIA measurement. The other one was dissolved in 2 mL sodium acetate buffer (50 mM, pH 5.1), containing 1 mg type H-1 β-glucuronidase/arylsulfatase l from *Helix pomatia* (Sigma–Aldrich, St. Louis, MO, USA) and 1 mg type II acid phosphatase from potatoes (Sigma–Aldrich). The enzymes in the buffer, will convert the conjugated ecdysteroids into free ecdysteroids, for recognition by the antibody. The reaction mixtures were kept at 37 °C for 24 h. Reactions were terminated by adding 100% methanol. Finally, all mixtures were dried as mentioned above. Samples were resuspended in 100 µL EIA buffer (0.4 M NaCl, 1 mM EDTA, 0.1% BSA in 0.1 M phosphate buffer) and stored at −20 °C until use.

Ecdysteroid levels were quantified with EIA method [39] using 20E EIA antiserum (Cayman Chemical Company Inc., Ann Arbor, MI, USA) and 20E acetylcholinesterase (AchE) (Cayman Chemical). The antiserum detects ecdysone (E), 20-hydroxyecdysone (20E) and other ecdysteroid metabolites including 2-deoxy-20-hydroxyecdysone and 2-deoxyecdysone. The EIA technique is based on the competition between the sample ecdysteroids and the ecdysteroid tracer for binding to the anti-ecdysteroid antibodies. The EIA plate was incubated with 100 µL EIA buffer, 50 µL samples, 50 µL 20E AchE tracer and 50 μL 20E EIA antiserum for 18 h at 4 °C. Later, the plate was emptied and rinsed 5 times with washing buffer. A coloration reaction was started either by adding of UHP (urea–hydrogen peroxide adduct, Sigma–Aldrich) and TMB (tetramethylbenzidine, Sigma–Aldrich) or alternatively with adding 200 µL of Ellman’s reagent (5,5′-dithiobis-(2-nitrobenzoic acid); DTNB) to each well, and then 5 µL tracer was added to total activity well. Optimum development was obtained using an orbital shaker equipped with a large flat cover to allow the plates to develop in the dark. All assays were performed in triplicates. Assay typically develops in 90–120 min. Absorbance was measured every 5 min at 412 nm for 1 h using a microplate reader (Epoch™; BioTek Instruments, Inc., Winooski, VT, USA). The standard curve was obtained with ecdysone (E) or 20E (Sigma-Aldrich, St. Louis, MO, USA) and results were expressed as E equivalents or 20E equivalents. For the hemolymph and ovary samples, 20E and E were chosen as standard respectively, since the main ecdysteroid in adult ovaries of *P. americana* is E [40] but 20E is abundant in female hemolymph [41].

### 2.9. Ecdysteroid (20E) Injection

Ten microliters of 20E (a kind gift of Dr. Karel Sláma, Institute of Entomology, Czech Academy of Sciences) were injected into the hemocoel of day 5 female roaches and the controls were injected with the same volume of control solvent using a Hamilton syringe (Hamilton, NV, USA). At this age, hemolymph 20E levels were low [41]. Ten µL aliquots of 20E were injected, after being dissolved in 10% ethanol and diluted so that the final concentration of EtOH was less than 0.1% and those of 20E were 0, 0.01, 0.05, 0.1, 0.25, 0.5, 1.0, 2.0, and 2.5 mM. The puncture made by the injection was sealed with the Aron Alpha (Toagosei, Tokyo, Japan). On the 7th day of the experiment BR-CA complex and fat body were isolated.

### 2.10. Statistical Analysis

**Figure 1:**
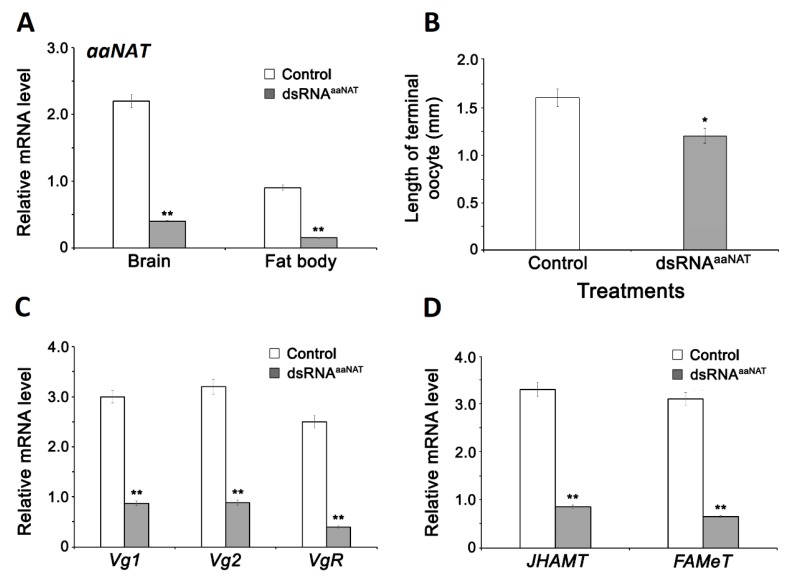
Aralkylamine N-acetyltransferase (aaNAT) acts in different aspects of vitellogenesis. (**A**) The relative expression of *aaNAT* mRNA in female brain and fat body compared to *actin* standard in adult females of *P. americana* 4 days post-injection with dsRNA^aaNAT^. (**B**) Oocyte length of adult females of *P. americana* 4 days post-injection with dsRNA^aaNAT^. (**C**) Expression of vitellogenin 1 and 2 (*Vg1* and *Vg2*) and vitellogenin receptor (*VgR*) mRNAs in female fat body of *P. americana* 4 days after dsRNA^aaNAT^ injection. (**D**) Expression of genes encoding JH acid methyltransferase (*JHAMT*) and farnesoate *O*-methyltransferases (*FAMeT*) in the brain of adult females of *P. americana* 4 days post-injection with dsRNA^aaNAT^. Data are presented as mean ± SEM. The significant differences are indicated by * (*p* < 0.05; **B**) or ** (*p* < 0.01; **A**, **C**, **D**), as compared to the controls, using Student’s *t*-test.

All results are presented as mean ± standard error of the mean (SEM). One-way analysis of variance (ANOVA) followed by post-hoc analysis using Duncan’s multiple range test (DMRT) or Tukey’s test were applied for comparisons among data. Student’s *t*-test was used for pairwise comparisons between the treated and control groups (data presented in Figure 1). In all cases, *p* < 0.05 were considered significant, unless otherwise stated. Statistical analysis was carried out using IBM SPSS Statistics Version 15.0. (Armonk, NY, USA).

## 3. RESULTS

### 3.1. aaNAT Acts in Vitellogenesis

Expression of *aaNAT* was knocked down by dsRNA injection. mRNA^aaNAT^ levels were significantly affected in the brain and fat body with knockdown efficiency of *aaNAT* was 78.10% and 84.59%, respectively after 4 days of injection (Figure 1A). The dsRNA treatments significantly reduced terminal oocyte compared with water-injected controls (Figure 1B). *aaNAT* knockdown led to about 70–90% reduction of *Vg1*, *Vg2 and VgR* mRNA compared with water-injected females (Figure 1C).

### 3.2. Effect of Indoleamines on Vitellogenin Synthesis

To determine whether monoamine directly influences Vgs and VgR synthesis or indirectly via JH synthesis, *JHAMT* and *FAMeT* expressions were investigated after dsRNA^aaNAT^ injection. The dsRNA^aaNAT^ treatment reduced *Vg1*, *Vg2,* and *VgR* expressions (Figure 1C). *JHAMT* or *FAMeT* levels in the brain-retrocerebral complex were many folds lower than water-injected controls (Figure 1D).

### 3.3. CCAP and sNPF Concentration in the Hemolymph after Injection of dsRNA^aaNAT^

**Figure 2:**
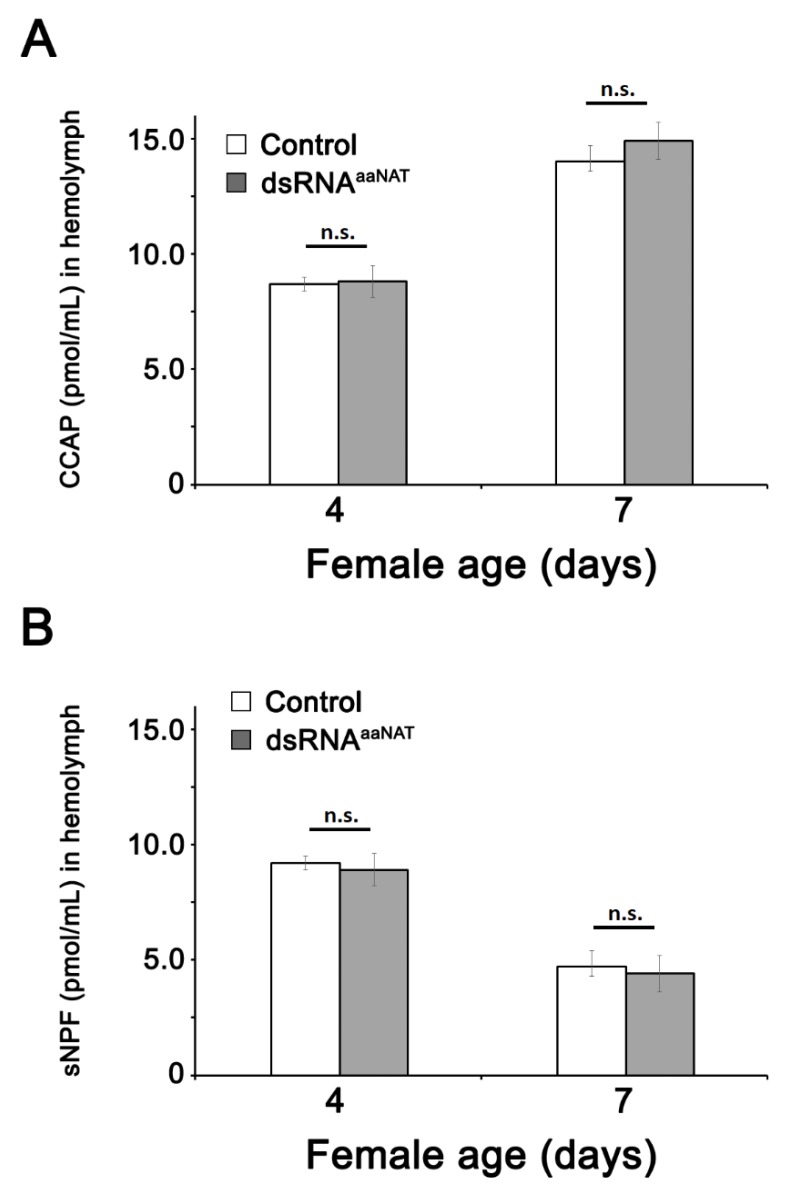
Concentrations of hemolymph crustacean cardioactive peptide (CCAP) (**A**) and short neuropeptide F (sNPF) (**B**) 4- and 7-days post-injection of *P. americana* adult females with dsRNA^aaNAT^. Data is displayed as mean ± SEM, (*n* = 25 individuals/condition). n.s., no statistically significant differences were observed between the tested groups using the Student’s *t*-test.

We examined whether the monoamine metabolic pathway is involved in CCAP/sNPF synthesis during vitellogenesis via the competitive ELISA assays using hemolymph samples. CCAP concentration increased with age in control and dsRNA-treated insects (Figure 2A), whereas sNPF concentration decreased with age, again with no dsRNA influence (Figure 2B).

### 3.4. Effect of CCAP and sNPF Injections on Ecdysteroid (20E) Titer in the Hemolymph and Ovary

**Figure 3:**
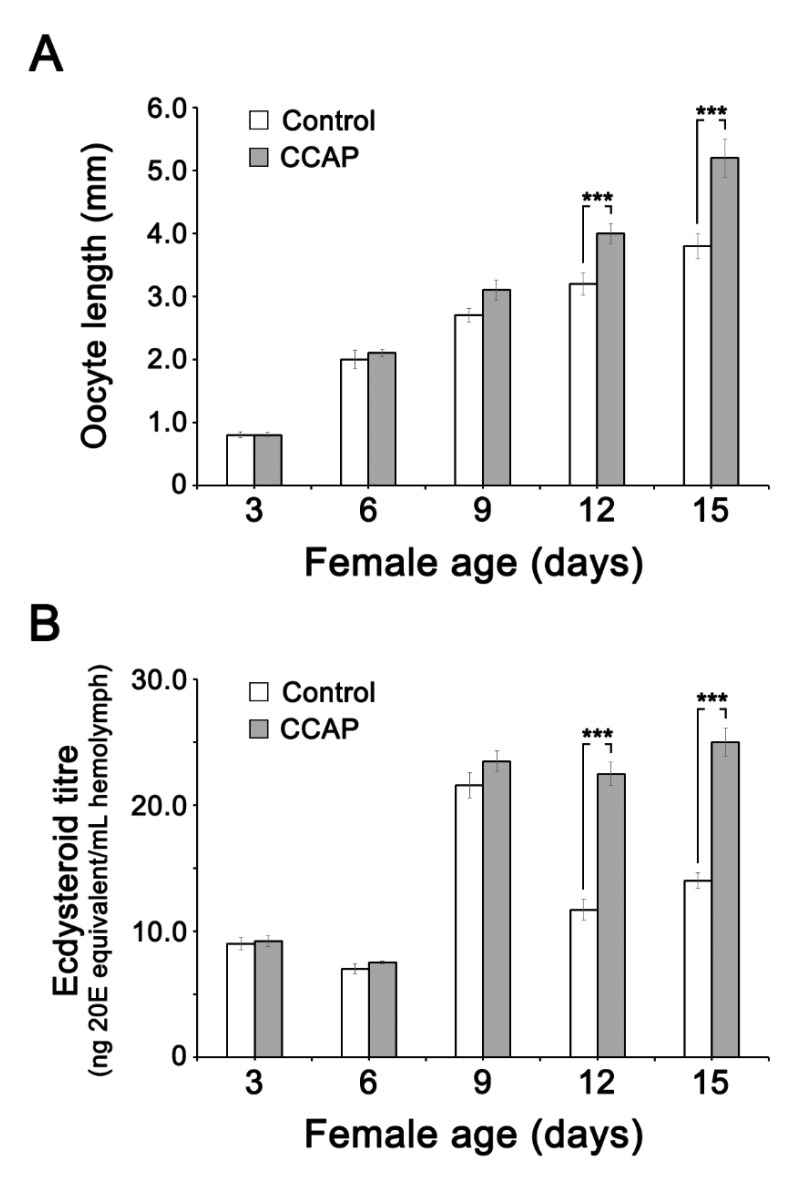
Effect of CCAP injection on oocyte growth and 20E titers in hemolymph. (**A**) Effect of CCAP injection on oocyte size in adult females. Adult females were injected daily with 10 pmol CCAP (dissolved in 5 μL of PBS), from day 1 to day 15 of the adult stage. Controls were injected with 5 μL PBS. (**B**) Effect of CCAP injections on ecdysteroid concentrations in the hemolymph. A total amount of 10 p moles of CCAP (in 5 μL of PBS) was injected daily into the hemocoel of female adults from day 1 to day 15. Control insects were injected with 5 μL of PBS. Ecdysteroid level was measured by the enzyme immunoassay (EIA). Data are expressed as mean ± SEM (*n* = 25 individuals/condition). ***: represent significant difference (*p* < 0.001) as compared to the control group, using Tukey’s test.

The oocyte maturation follows female age (Figure 3A). It is accompanied by an increase in ecdysteroid titer (Figure 3B). The influence of CCAP injection on 20E titer was quantified via the EIA at ages 3, 6, 12 and 15 days after adult emergence (Figure 3B). The effect of CCAP injection was significant during the second half of vitellogenesis, i.e., day 12 and 15. 20E showed peak on day 9 but declined thereafter in normal course, but 20E titer was maintained high if the cockroaches were injected with CCAP. The oocytes continued to grow with or without CCAP injection, but CCAP injection stimulated oocyte growth more than water-injected control (Figure 3A). Effect of CCAP injection, measured at day 15 post-injection, on free and total ovarian ecdysteroid concentrations is given in Figure S1.

**Figure 4:**
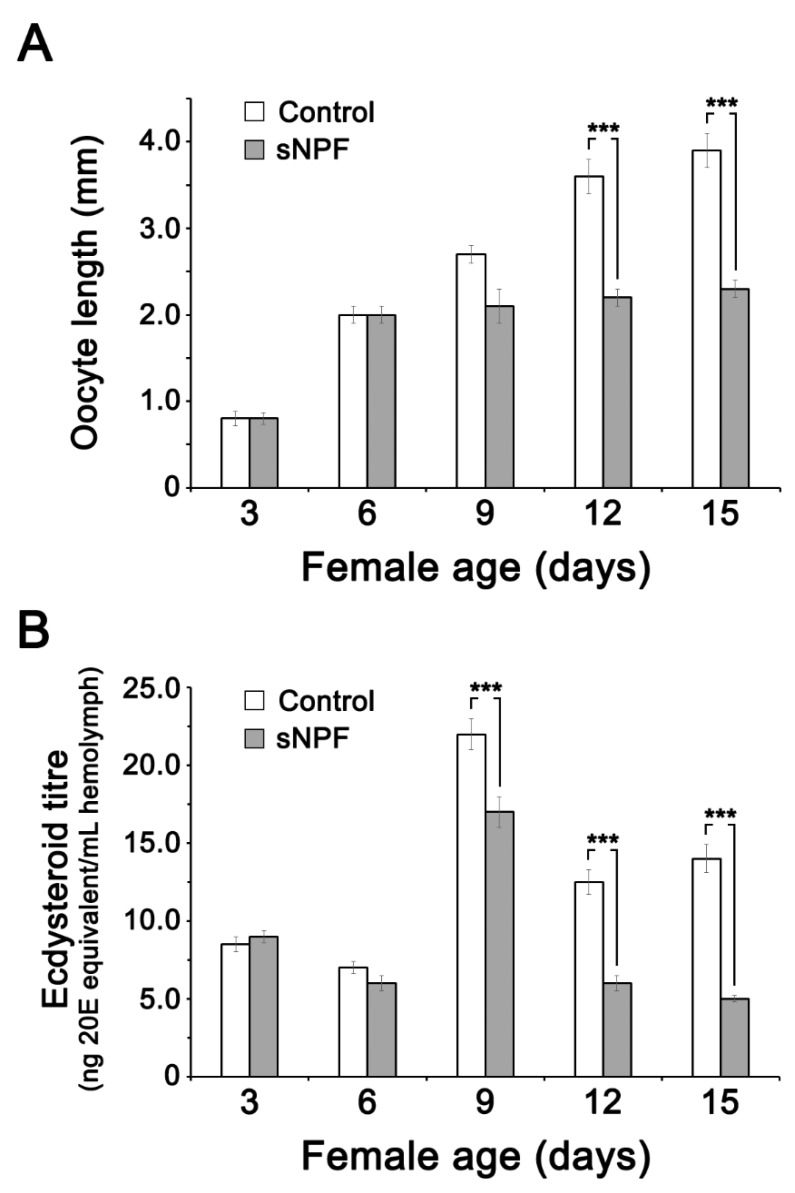
Effect of sNPF treatments on oocyte size and 20E titers in hemolymph. (**A**) Effect of sNPF injection on oocyte size in adult females. Adult females were injected daily with 10 pmol sNPF (dissolved in 5 μL PBS), from day 1 to day 15 of the adult stage. Control insects were injected with 5 μL PBS into the hemolymph. (**B**) Effect of sNPF injections on ecdysteroid concentrations in the hemolymph. A total 10 pmol of sNPF (in 5 μL of PBS) was injected daily into the hemocoel of female adults from day 1 to day 15. Control insects were injected with 5 μL of PBS. Ecdysteroid level was measured by the EIA. Values are displayed as mean ± SEM (*n* = 25 individual/condition). ***: significantly different (*p* < 0.001) compared to the controls, using Tukey’s test.

Meanwhile, sNPF injections suppressed oocyte maturation significantly at 12 and 15 days after injection (Figure 4A). The effect of sNPF injection on 20E titer were quantified at ages 3, 6, 12 and 15 days after adult emergence (Figure 4). sNPF treatments significantly reduced hemolymph 20E titers at 9–15 days after injection (Figure 4B). Effect of sNPF injection, measured at day 15 post-injection, on free and total ovarian ecdysteroid concentrations is given in Figure S2.

### 3.5. Effect of Neuropeptides (CCAP and sNPF) on JH Synthesis Pathway during Vitellogenesis

**Figure 5:**
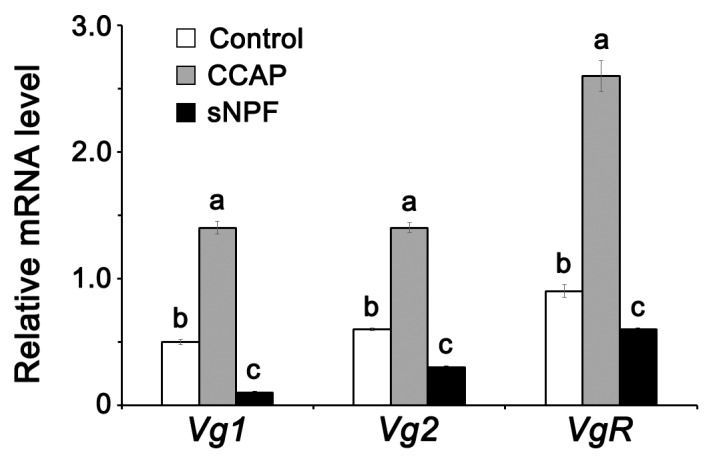
*Vg1*, *Vg2,* and *VgR* expression in the fat body of normally fed adult females of *P. americana* 4 days post-injection with CCAP and sNPF. Data are presented as mean ± SEM. Means followed by different letters are significantly different (*p* < 0.05), using Duncan’s multiple range test.

**Figure 6:**
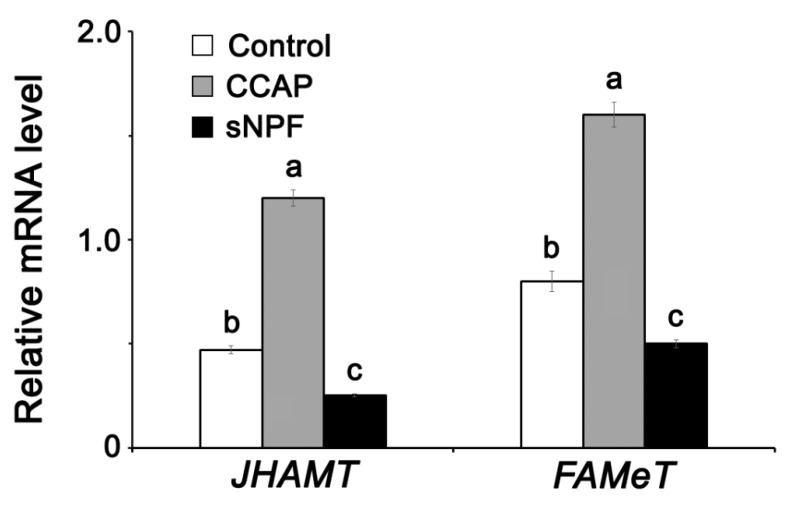
*JHAMT* and *FAMeT* expression in the fat body 4 days after CCAP and sNPF injection into normally fed *P. americana*. Data are presented as mean ± SEM. Means followed by unlike letters are significantly different (*p* < 0.05), using Duncan’s test.

CCAP and sNPF injections led, significantly and respectively, to an increase and a decrease of mRNAs encoding Vg1, Vg2, and VgR in the fat body in comparison to controls (Figure 5). Similarly, CCAP and sNPF treatments led, significantly and respectively, to an increase and a decrease of *JHAMT* and *FAMeT* expression compared to controls (Figure 6).

### 3.6. Ovarian Cellular Distribution of PaVgR Protein 

**Figure 7:**
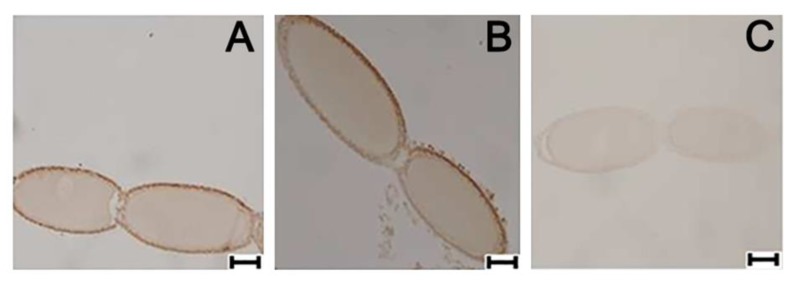
Immunocytochemical detection of VgR in *P. americana* ovaries from virgin female by immunofluorescence. Control (**A**), CCAP injected (**B**) and sNPF injected (**C**) of normally fed insect 4 days after injection. Scale bar = 200 μm.

To determine the cellular distribution, reflecting the above RNA transcriptional pattern, the presence of VgR protein was recorded at late previtellogenic stage by immunocytochemical localization in the terminal oocytes (Figure 7A). CCAP treatments led to visible increases in VgR protein compared to control and to sNFP treatments (Figure 7B). sNPF treatments suppressed VgR expression compared to controls (Figure 7C).

### 3.7. Dose Responses by Injection of 20E on Vitellogenesis

**Figure 8:**
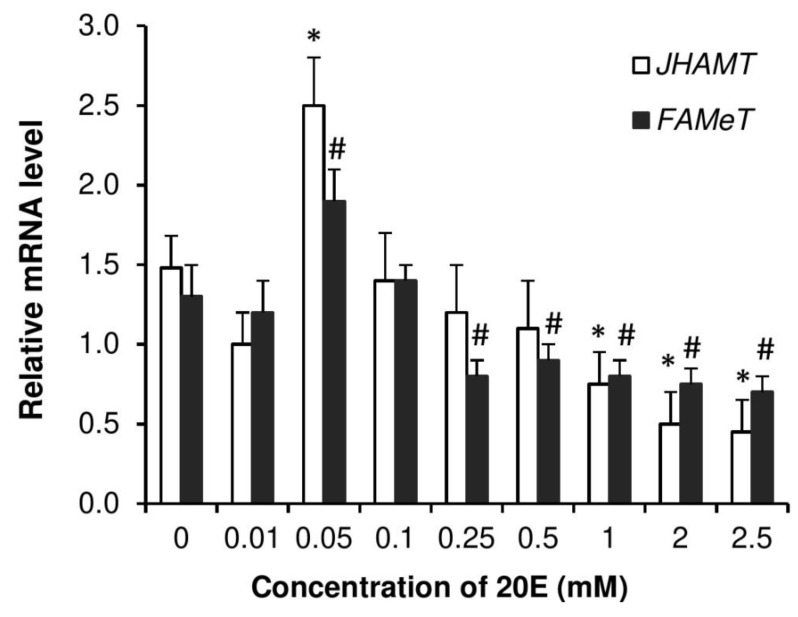
Dose response for induction by 20E. Expression of *JHAMT* and *FAMeT* in the brain of adult *P. americana* females 4 days after injection with 10 µL of the different given concentrations of 20E. Control roaches were injected with 10 µL of ca. 0.1% EtOH solvent. Data are presented as mean ± SEM. Columns with symbols (*: *JHAMT*; #: *FAMeT*) are significantly different at *p* < 0.05, compared to 0 concentration, using Tukey’s test.

**Figure 9:**
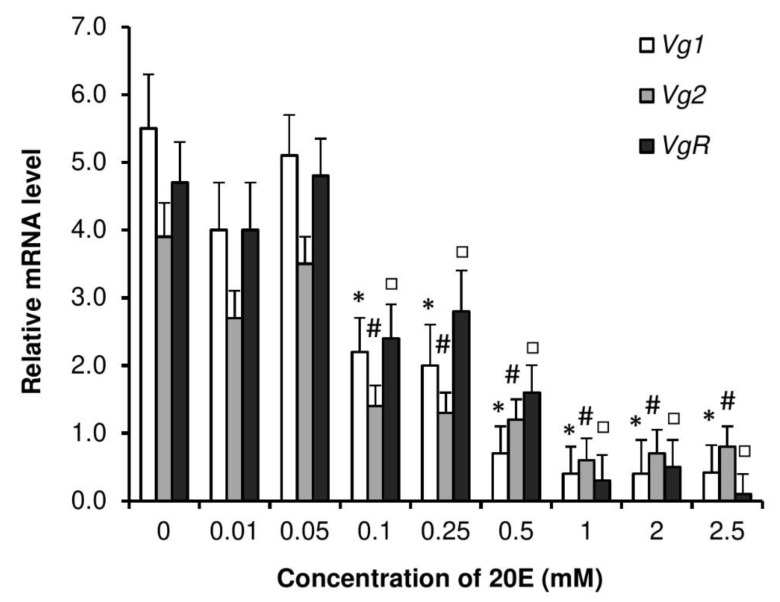
Dose response for reduction by 20E. Expression of *Vg1*, *Vg2,* and *VgR* mRNA in *P. americana* female fat body 4 days after injection with 10 µL of the different doses of 20E. Control roaches were injected with 10 µL of ca. 0.1% EtOH solvent. Data are shown as mean ± SEM. Columns with symbols (*: *Vg1*; #: *Vg2*; □: *VgR*) are significantly different at *p* < 0.05, compared to 0 concentration, using Tukey’s test.

To observe whether increase in 20E levels has any effect on JH metabolic pathway in *P. americana*, transcriptional levels of *JHAMT* and *FAMeT* were measured in 20E-injected females. Accumulations of mRNAs encoding *JHAMT* and *FAMeT* peaked at 0.05 mM and decreased with increased and decreased concentrations of injected 20E (Figure 8). Similarly, Figure 9 shows that 20E treatments led to nearly dose-dependent decreases of mRNAs encoding Vg1, Vg2 and VgR in virgin females.

## 4. Discussion

aaNAT operates in a variety of behavioral, developmental, physiological, reproductive and metabolic processes suggesting that multiple aaNAT forms occur in insects [28]. High levels of aaNAT activity were detected in female colleterial glands and the pH profile changed with oocyte maturation when TN and 5-HT were injected into *P. americana* [25]. Our data show that dsRNA^aaNAT^ injection led to substantial gene silencing which triggered significant physiological changes, i.e., it reduced both oocyte length and transcription of *Vg1*, *Vg2* and *VgR*. The treatments decreased transcription of *JHAMT* and *FAMeT*. However, the dsRNA treatments did not influence CCAP, nor sNPF concentrations. Earlier, we have shown that indoleamines affected the expression of *JHAMT* and *FAMeT* in the *P*. *americana* Br-CA complex where 5-HT inhibited *JHAMT* and *FAMeT* transcription, while TN and NATN stimulated their transcription [26]. Biogenic amines act as neurohormones regulating insect gonadotropins, i.e., JH and 20-E, both in vitro and in vivo [42].

CCAP treatments increased oocyte length, and both hemolymph and ovarian 20E titers. Contrariwise, sNFP decreased oocyte lengths, and both hemolymph and ovarian 20E titers. CCAP, increased *Vg1* and *Vg2*, and *VgR* gene expression and oocyte VgR protein but sNPF decreased *Vgs* and *VgR* expression. CCAP stimulated expression of *JHAMT* and *FAMeT,* while sNPF suppressed them. 20E treatments exerted the inhibitory influence over *Vg/VgR* transcription but had a peak at 500 pmol in *JHAMT* and *FAMeT* transcription.

These peptides altered vitellogenesis but the mechanism by which they may exert their action may be either (1) directly on the oocyte membrane, i.e., via endocytosis, (2) via the fat body, i.e., Vg synthesis, (3) the CA, i.e., JH synthesis, (4) the prothoracic gland, i.e., ecdysone synthesis, (5) peripheral organ such as the midgut, i.e., monooxygenation of ecdysone, (5) the follicle cells, i.e., intercellular junction or (6) the brain, i.e., release of proper allatotropin (AT) or allatostatin (AS). Redundant pathways may be required. To determine the exact mechanism, further future experiments remain to be conducted such as examining peptide functions in allatectomized females. Also, monitoring syntheses of JH in the CA and Vgs in the fat body after injection of peptides into decapitated females. As well as observing the growth of gonad cultured independently from the brain, the CA and the prothoracic glands in response to peptides and monoamines application.

The major interest lies in the mechanisms of interaction among these factors/pathways. aaNAT is required for oocyte development, seen in oocyte lengths and in transcription activities of *Vg*s and *VgR*. aaNAT and its monoamine products may regulate JH biosynthesis, but not via CCAP or sNPF, because dsRNA^aaNAT^ reduced expression of genes implicated in JH biosynthesis but did not influence CCAP or sNPF. We retrieved two aaNAT gene sequences tentatively called aaNAT_A_ and aaNAT_B_ that had different optimal pH at acidic and basic side, respectively [29,43]. The sequence used here for RNAi corresponds to aaNAT_A_. More precise matching between aaNAT and monoamine species require further studies. Yet, at least, we confirm that aaNATs and their products are involved in vitellogenesis in *P*. *americana*. The CA of *M. sexta* has dopaminergic innervation and dopamine possibly plays a critical role in the JH biosynthesis [44].

CCAP increased oocyte length, *Vg1/Vg2* and *VgR* transcriptions, and 20E titers. 20E treatments reduced mRNAs of both *JHAMT* and *FAMeT* and reduced mRNAs of *Vg*s and *VgR*. The influence of 20E treatments were recorded at 4 days post-injection (pi). The Vg gene begins to be expressed in the 2-day-old adult female fat body cells [24]. On the other hand, CCAP treatments significantly affected oocyte length and 20E concentrations from days 12-15 pi. Conventionally it has been considered that the female reproductive maturation in most insects depends on JH [45]. JHAMT and FAMeT are critical for JH biosynthesis during vitellogenesis, and in promoting oocyte maturation [46,47]. JHAMT converts JH acids or inactive precursors of JHs to active JHs at the final step of JH biosynthesis [48], whereas FAMeT catalyzes the formation of methyl farnesoate from farnesoic acid in the JH biosynthetic pathway [49]. However, an unlikely role of FAMeT in JH metabolism remains a matter of debate [50,51,52,53,54]. Effects of CCAP and sNPF on 20E production are more intensely expressed in the second half of vitellogenesis. Unique regulation may occur in vitellogenesis, possibly JH-dependent first step and 20E- and peptides-dependent second step as Vg accumulation in oocytes undergoes two steps in *P. americana* [2,55]. The expression of *Vgs* has a slight time lag and there may be a need for distinct hormonal regulations, either timewise or gene-wise.

Some neuropeptides have prothoracicotropic, allatotropic and allatostatic effect on hormonal regulation [56,57]. CCAP, for example, was stimulatory, while sNPF had an inhibitory effect on food uptake in *P. americana* [17,58]. In response of the midgut to food intake, CCAP released from the midgut epithelium induces a positive feedback loop to stimulate massive release of CCAP from the brain to the hemocoel to stimulate massive release of digestive enzymes from the midgut but it also acts on the same midgut secretory cells to shut down the synthesis and release of sNPF in the autocrine negative feedback loop [17]. Such a complex endocrine network may extend to AT/AS peptide network. CCAP may induce the release of AT proper and sNPF may release AS proper. sNPF was reported to have JH-inhibiting effects [59,60] and the expression pattern of sNPF in our study is similar to that of ASs [61]. In locust, sNPF indirectly affected the synthesis of vitellogenin via JH synthesis route [62]. In current experiments, we tried to explore the allatoregulatory roles of CCAP-AT and sNPF-AS in the regulation of reproduction in virgin *P. americana.* CCAP stimulated oocyte development and also upregulated the 20E level in the hemolymph and ovary; on the other hand, sNPF inhibited oocyte development and downregulated the 20E titer in the hemolymph and ovary.

In mosquito, immunocytochemical evidence revealed that Vg binds with VgRs in between cortex of oocyte and at the base of microvilli in the vitellogenic ovaries [63], it was supported by immunofluorescent labelling of VgR in the same location [64]. Consistent with previous evidence, PaVgR is localized in the terminal oocyte surface [3,65].

We also found that injection of 20E affected *JHAMT/FAMeT* transcription and exhibited reduction of mRNA of these enzymes in dose-dependent manner showing maximum activity at 0.05 mM dose, i.e., 500 pmol. Consequently, *Vg*s and *VgR* expressions were also inhibited by 20E injection. In *D. melanogaster*, the results of topical application of JHA (JH analogue) and injection of 20E on vitellogenesis revealed that the maturation of vitellogenic oocytes, including production of yolk proteins and their uptake by the oocytes is stimulated by JH, while 20E regulates early vitellogenic stages of the oocyte maturation [66]. The authors also propose that for the normal regulation of oogenesis in *Drosophila*, a proper balance between JH and 20E is of a paramount importance [66].

Several lines of evidence also supported the above results in adult *P. americana* that ovarian ecdysteroids are involved in inhibition of JH biosynthesis like in *D. punctata* [67] and *Blattella germanica* [68]. The effect seems to be indirect because the presence of 10^−5^ M 20E fails to inhibit JH biosynthesis in cultured CA from *D. punctata* [69]. CCAP increased 20E level in the 2nd vitellogenic stage, while sNPF increase 20E level in the second half of vitellogenic cycle then decrease later. We reported occurrence of *JHAMT*/*FAMeT* in pre-vitellogenic stage, which means that Vg biosynthesis needs a proper balance of JH and 20E. We suggest that these changes in 20E titer are associated with the initiation of reproduction and that, if allatoregulatory neuropeptides are involved in controlling 20E biosynthesis, their presence and/or activity at these times should be consistent with the changes in 20E levels. Nevertheless, the fact that we have detected significant stage-specific changes of 20E titer in the hemolymph and ovary of *P. americana* virgin female, suggests that these secretions may indeed be regulated by neuropeptide ATs and/or ASs or their alias. Based on the current experiments, we propose that the CA of *P. americana* are continually active in producing JH (possibly by continuous stimulation by CCAP-AT). Nevertheless, the results of our experiments indicate that both neuropeptides and indoleamines are indeed implicated in the modulation of CA activity in this cockroach during vitellogenesis.

**Figure 10:**
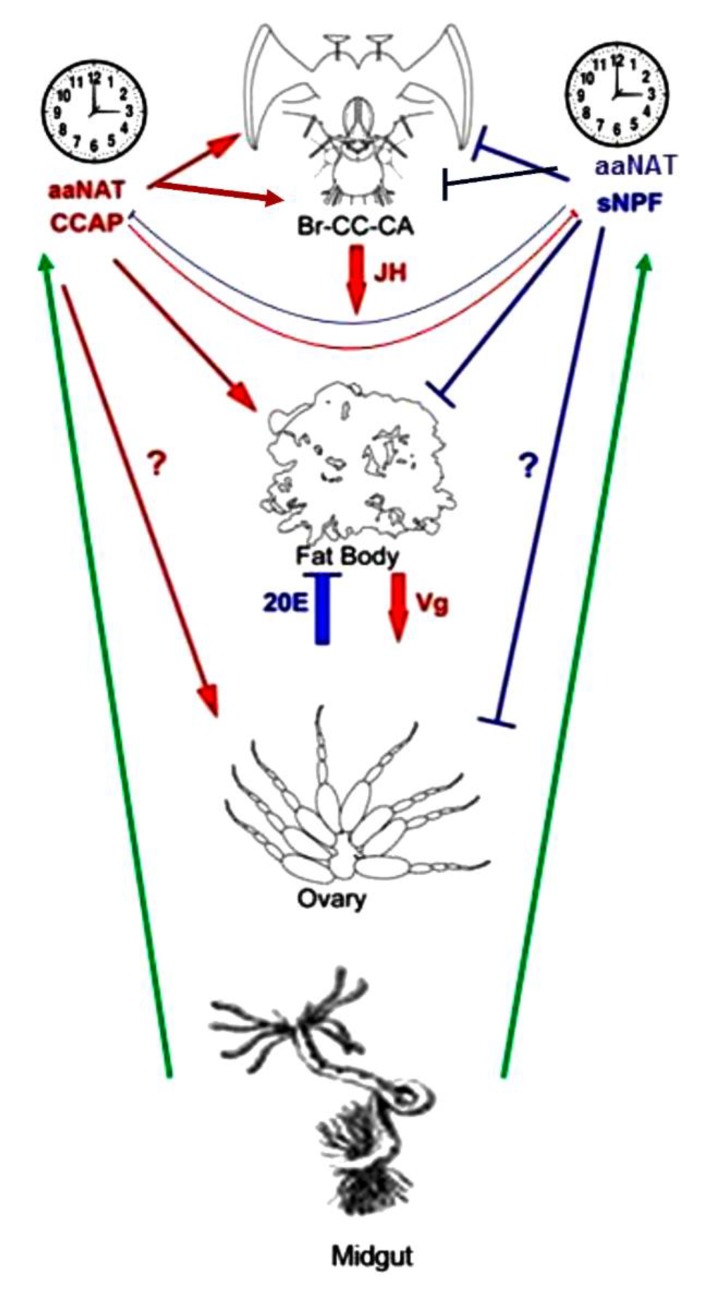
The “physiological wormhole model” for control of insect reproduction. Three distinct routes of neuroendocrine pathways, running in parallel or interactive or linearly connected. Probably non-endocrine regulatory pathways operate to regulate reproductive timing, associative behaviors, metabolic supports, JH/20E, CCAP/sNPF and monoamines as well as the biological clock, longevity, and cell cycle machinery.

This study clearly demonstrated an interlocked feedback system comprising of cerebral organs and digestive system even in reproductive regulation. We have relied on a more pleiotropic view to the hormonal regulation of reproduction; that is JH practically affects all end targets, from the fat body, brain, retrocerebral complex, gonad, to oocytes and so on. The present data point out the need for an alternative view of ‘complex’ regulation of reproduction rather than the classical one dependent on the hormonal milieu. It can be outlined as “a network integrating various demands of the terminal organs”, as illustrated in Figure 10, that we might call it “the physiological wormhole model”. Here at least, JH/20E, enteroendocrine neuropeptides, and monoamines convey important messages from peripheral organs, brain, midgut, fat body, gonads, retrocerebral complex, and other endocrine organs and sensory systems. Taking into account that logistic support for reproduction acquires the close association of fat body and midgut systems particularly for cruiser type foragers like cockroaches, unlike caterpillars that stay on the same host plants without frequent moves.

## 5. Conclusions

Considering all the above information, we conclude that (1) aaNAT is involved in vitellogenesis via changed balance of monoamine neurotransmitters. dsRNA^aaNAT^ injection downregulated JH synthetic pathway and oocyte growth. (2) sNPF downregulated JH synthetic pathway and vitellogenesis while CCAP upregulated them. VgR was regulated in the same way and direction. (3) CCAP injection stimulated 20E synthesis and oocyte growth, while sNPF suppressed them particularly in the second half of vitellogenesis. (4) We interpret our results to show that 20E might play an important role (including regulation of JH titer) in vitellogenic process, but the function of 20E during oocyte maturation is complex. Our results show that the adult ecdysteroid (20E) is involved in inhibition of JH biosynthetic pathway. (5) The results clearly demonstrated crosstalks among three hormonal regulatory axes in reproduction. Functional interactions between CCAP/sNPF and AT/AS, and interactions between indolamines and catecholamines require further study.

## Figures and Tables

**Table 1:** A list of primers used in the experiments.

Gene	Primer Sequence (5′–3′)
*aaNAT*-FT7	TAATACGACTCACTATAGGGAGATAATGGCAGTATCCAGAAC
*aaNAT*-FT7	TAATACGACTCACTATAGGGAGAGATATTATGCGCACTTCTAC
*aaNAT* qpcr F	TGTGTTTCAACCAGCTCTGC
*aaNAT* qpcr R	AACTTCCACTCGTAGTGGTTCC
*Vg1* Forward	CCAGACATTATCAGACCTCCAGTAG
*Vg1* Reverse	TGTAGGTTTGAAGGCCACAATAGTA
*Vg2* Forward	CTTACACGAGGTCGCAAATCAG
*Vg2* Reverse	CTGTCATGTGATACGTGTCTTTGAG
*VgR* Forward	TGTCTTGTGAAGATGGATTTGTGTG
*VgR* Reverse	CACTGTTGTCTCCACAATCATCAAA
*JHAMT* Forward	GAAGCTCTCATAGTATTCGTGGC
*JHAMT* Reverse	AGGATCTTCTGACTGATGGTAGG
*FAMeT* Forward	ACTGTATGTAGGACGGGCAAAG
*FAMeT* Reverse	CCAGTCAGCACCTCATATTCAG
*Actin* Forward	TGAATCCTAAGGCCAACAGG
*Actin* Reverse	ACCGGAATCCAGCACAATAC

The T7 RNA polymerase promoter sequence is underlined.

## Supplementary Materials

The following are available online at https://www.mdpi.com/2075-4450/11/3/155/s1, Figure S1: Effect of CCAP injection on ovarian ecdysteroid concentrations. Figure S2: Effect of sNPF injection on ecdysteroid concentrations in the ovaries.
