# Supplementary material for: Crosstalk among Indoleamines, Neuropeptides and JH/20E in Regulation of Reproduction in the American Cockroach, Periplaneta americana
Source: Insects. 2020 Mar 1;11(3):155. doi: 10.3390/insects11030155 (PMC7143859; doi:10.3390/insects11030155)
Supplement: Supplementary file 1 [file insects-11-00155-s001.zip › Figure S2.pdf]

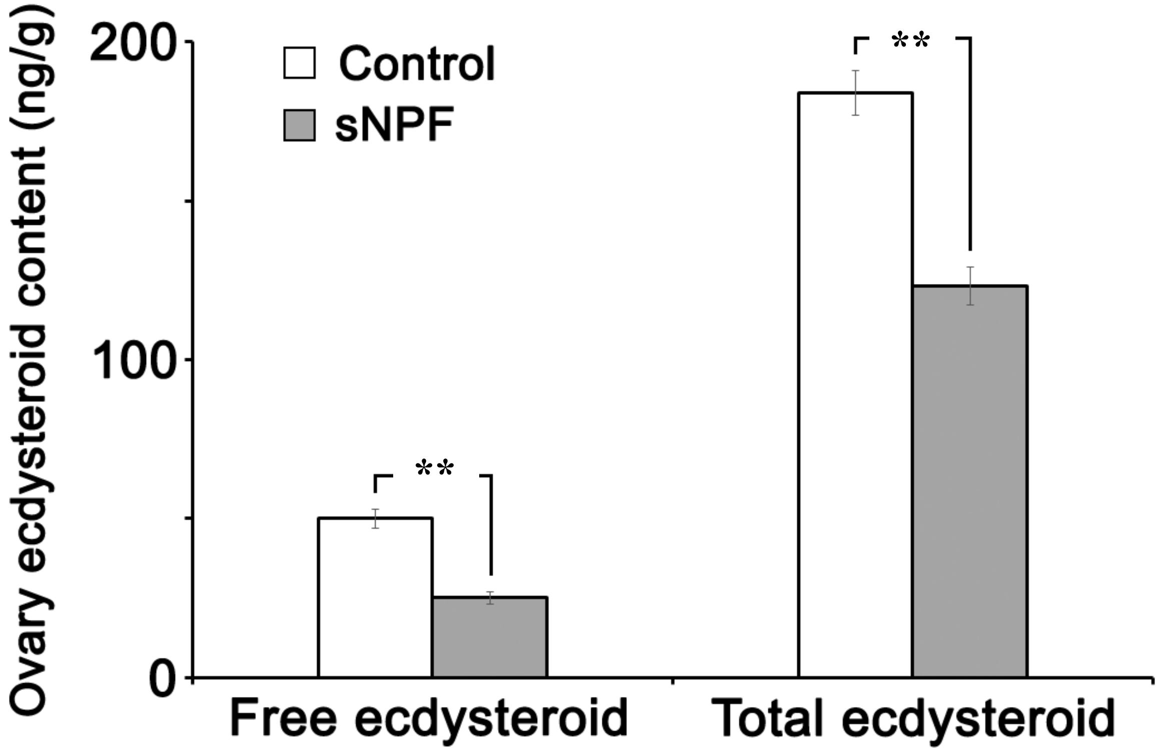

Figure S2. Effect of sNPF injection on ecdysteroid concentrations in the ovaries. A total amount of 10 pmol of sNPF (in 5 μL of PBS) was injected daily into the hemocoel of female adults from day 1 to day 15. Control insects were injected with 5 μL of PBS. On day 15, ovaries were dissected and ecdysteroid level was measured by the EIA. Data are expressed as mean ± SEM (n=25 individuals/condition). **: represent significant difference (p <0.01) as compared to the control group, using Tukey's test.
